# Research Progress on the Application of Plant Growth Regulators in the Rapid Propagation of Jujube by In Vitro Culture

**DOI:** 10.3390/plants14193012

**Published:** 2025-09-29

**Authors:** Bochao Yang, Zhi Luo, Xingyu Zhu, Yinzhong Ji, Quanhui Ma, Fenfen Yan

**Affiliations:** 1 The National and Local Joint Engineering Laboratory of High Efficiency and Superior-Quality Cultivation and Fruit Deep Processing Technology of Characteristic Fruit Trees, College of Horticulture and Forestry, Tarim University, Alar 843300, China; 2 Xinjiang Production and Construction Crops Key Laboratory of Protection and Utilization of Biological Resources in Tarim Basin, Tarim University, Alar 843300, China

**Keywords:** auxin, cytokinin, gibberellin, jujube, in vitro culture rapid propagation

## Abstract

Jujube (*Ziziphus jujuba* Mill.) is an important economic fruit tree in China, and its in vitro culture technology is the key to achieving large-scale seedling cultivation. PGRs (Plant growth regulators) play a central regulatory role in all stages of jujube micropropagation, including explant initiation, proliferation, and rooting. This article provides a comprehensive overview of recent advances in in vitro culture of jujube, with a focus on the recommended exogenous phytohormone ratios, their effects, and underlying regulatory mechanisms across distinct varieties during the key stages such as in vitro culture, shoot proliferation, and root formation. The primary culture of most jujube varieties usually employs the MS medium, and it is recommended that auxin and cytokinin be used in combination. During the initial cultivation stage, the use of NAA (1-naphthaleneacetic acid) or IBA (indole butyric acid) is recommended at concentrations ranging from 0.1 to 1.0 mg/L. At the same time, 6-BA (6-benzylaminopurine) is suggested, with a concentration range of 0.5 to 2.5 mg/L. In the subculture multiplication of most jujube varieties, MS medium is used, and auxin (such as NAA, IBA), and TDZ (thidiazuron) and cytokinin (e.g., 6-BA) are used in combination. The recommended concentration range for auxin remains between 0.1 and 1.0 mg/L, and for cytokinin 6-BA between 0.5 and 2.5 mg/L, while the recommended concentration of TDZ is suggested to be below 0.01 mg/L. Rooting induction for most jujube varieties has predominantly been achieved using 1/2 MS medium, with growth regulator concentrations typically ranging from 0.5 to 3.0 mg/L.

## 1. Introduction

Jujube (*Ziziphus jujuba* Mill.), native to China, is one of the oldest cultivated fruit trees. It is recognized as one of the ‘Five Fruits’ alongside plums, chestnuts, peaches, and apricots—it also ranks as one of the top five dried fruit trees [1,2]. At present, grafting serves as the primary method for jujube propagation. Nevertheless, this approach presents significant limitations: there are seasonal restrictions, slow-healing graft unions are susceptible to pests and diseases, and its survival rate is easily restricted by the environment, grafting time and technical proficiency [3,4]. When grafting technology touches the bottleneck of efficiency and scale, in vitro culture, with its controlled environment and strong expansion capacity, opens up a new path for jujube propagation, which is not subject to geographical, temporal or spatial constraints, and can utilize the totipotency of the cells to cultivate a complete plant, realize the rapid breeding of fine varieties, accelerate the breeding process, and also realize the preservation of fine varieties of germplasm resources, and provide an effective way for the rapid propagation of good jujube varieties [5]. Within the system of in vitro propagation, exogenous PGR has some effect on plant propagation. This review described possible exogenous hormone ratios for various jujube varieties during the induction, proliferation, and rooting stages, and discusses their associated hormonal effects and regulatory mechanisms. The objective is to provide a reference for in vitro rapid propagation and molecular breeding in jujube.

## 2. The Effect and Regulation Mechanism of Plant Growth Regulators on Plant Growth

### 2.1. The Role and Regulatory Mechanism of Auxin

Despite being applied in low concentrations, plant growth regulators are instrumental in regulating different stages of plant vitro culture and rapid propagation. Commonly employed classes of PGRs encompass auxins, cytokinins, and gibberellins. Auxin defining characteristic is the ability to undergo polar transport, establish concentration gradients. It is the first to be discovered in plants, is a fundamental regulator of physiology. It governs processes such as seed dormancy, germination, cell division, bud differentiation, and root development by altering internal hormone concentrations and modulating associated enzymatic activity [6,7,8]. It has been shown that the ARF transcription factors in the auxin signaling pathway can positively regulate seed dormancy, and they can also respond to the growth requirements during seed germination [9]. During seed germination, Exogenous auxin (e.g., IAA) positively influences the biosynthesis and signal transduction pathways of abscisic acid (ABA) while exerting a negative regulatory effect on those of gibberellin (GA) [10,11]. Furthermore, exogenous auxin has an effect on root development by mediating the unidirectional canalization of endogenous auxin, which promotes the formation of adventitious roots and lateral roots, enhances root density and length, and modulates root morphological structure. Exogenous auxin can also modulate endogenous levels of auxin and gibberellin, thereby facilitating the formation of adventitious roots [12]. Low concentrations (e.g., 10 nM) of exogenous auxin significantly promote root growth and development. In contrast, excessively high concentrations inhibit root system growth by perturbing the spatial expression patterns of Auxin Response Factors (ARFs), which act as critical mediators in a cell-type-specific manner [13]. Additionally, auxin may participate in the regulation of adventitious root growth by inducing H_2_O_2_ synthesis [14]. Exogenous auxin also promotes cell division by modulating the metabolism and balance of endogenous hormones, and further regulates the expression of cell division-related genes through signal transduction pathways. Studies have shown that exogenous auxin can stimulate the accumulation of endogenous cytokinins, thereby facilitating cell division [15]. In Arabidopsis, auxin is also involved in the reconstruction of the shoot apical meristem by regulating the expression of the WUS gene [16]. In addition, auxin can further affect cell division by regulating the expression of downstream genes through ARRs (e.g., type B ARRs) [17].

The primary mechanism of auxin action involves a unique nuclear signal transduction pathway. It is dominated by TIR1/AFB-Auxin-Aux/IAA-ARF, through which both endogenous and exogenous auxins can function [18]. In this transduction pathway, TIR1/AFB functions as the primary receptor. Upon binding with auxin, it initiates the ubiquitination and degradation of Aux/IAA repressor proteins. This process leads to the release of downstream auxin response factors (ARFs) that kickstart signal transduction. ARF transcription factors play a central role in mediating auxin signals by controlling gene expression through binding to target gene promoters [19]. Auxin has long been recognized as a master regulator of plant growth and development, orchestrating processes such as embryogenesis, organogenesis, and tropisms [20]. However, the types of action of auxin are not monolithic but exhibit remarkable specificity [21]. This specificity operates on at least two levels: (i) Cell-type specificity. It is important to note that the auxin response is cell-type specific, largely determined by the spatial expression and distinct combinations of ARFs and Aux/IAA proteins. Acting as suppressors, Aux/IAA proteins hinder ARF activity through interaction, thereby modulating gene expression in response to fluctuations in auxin levels [22,23,24,25]. Another potential auxin-binding protein, ABP1, expedites signal transmission to regulate the transcription of early auxin-responsive genes [26]. Furthermore, certain atypical Aux/IAA proteins may participate in auxin signal responses [27]. For instance, in the auxin-TMK1-IAA32/34 signaling pathway, the absence of Domain I/II enables specific interaction with and phosphorylation of two non-classical transcriptional repressors from the auxin or Aux/IAA family (IAA32 and IAA34), thereby influencing ARF transcription factors [28]. And (ii) Auxin-type specificity, Although various synthetic auxins (plant growth regulators) can mimic the core functions of natural auxins, such as indole-3-acetic acid (IAA)—including the promotion of cell elongation—subtle differences in their chemical structures lead to significant variations in their uptake, transport, metabolic stability, and affinity for different receptor proteins in plants. These differences ultimately result in distinct and specific physiological effects, potency, and duration of action [29].

### 2.2. Role of Cytokinins and Regulatory Mechanisms

Cytokinins, primarily composed of adenine derivatives, stimulate cell division and differentiation in specific cell types through a well-defined phosphorelay signaling pathway [30]. While endogenous cytokinins are fundamental regulators of various physiological processes—including cell division, differentiation, seed germination, root development, and floral organ development [31,32]—the application of exogenous cytokinin allows for the manipulation of these processes. For instance, exogenous cytokinin can significantly promote seed germination in certain plant species by targeting embryonic cells. Under in vitro culture conditions, specific cytokinins such as 2iP riboside have been shown to effectively enhance germination, while others exhibit limited effects [33]. This specificity may be related to the differential activation of cytokinin receptors in seed tissues, leading to the downregulation of ABI5 gene expression and inhibition of ABA signaling, thereby releasing seed dormancy. The effects of exogenous cytokinins (e.g., 6-BA, KT) are highly concentration-dependent and vary among species. An appropriate concentration can improve germination rate and seed vigor, whereas excessively high concentrations can inhibit germination [34]. For example, the optimal germination effect for Callicarpa formosana seeds was observed under treatment with 10 mg/L 6-BA, with higher concentrations suppressing germination [35]. In root development, the role of cytokinin exemplifies cell-type-specific regulation. Exogenous cytokinin primarily inhibits primary root elongation by targeting the meristematic cells in the root apex. Conversely, it promotes lateral root initiation and root hair formation by modulating hormone transport and signaling in pericycle and epidermal cells, respectively [36]. This regulatory role is associated with the modulation of auxin transport carriers such as OsAUX1 [37]. Furthermore, in shoot tissues, exogenous cytokinin application stimulates bud proliferation and formation from axillary meristem cells, delays leaf senescence by acting on mesophyll cells, and promotes stem growth. This leads to an increase in the number of stems per plant, stem length, and leaf area, ultimately leading to higher yield [38,39,40]. For example, in triploid lilies, cytokinin promotes bulbil formation by specifically regulating the expression of *LlWOX11* in the bulb scale meristem cells [41]. In bananas and Acanthopanax, culture media containing 6-BA or meta-topolin (a new type of cytokinin) significantly enhance stem elongation and lateral shoot proliferation by activating cytokinin-responsive genes in the shoot apical meristem and axillary buds [42,43].

The cytokinin signaling pathway involves intricate molecular mechanisms encompassing signal perception, transduction, and downstream gene expression regulation. Cytokinins initiate signal transduction by binding to histidine kinases (AHKs), including AHK2 and AHK3, which subsequently phosphorylate phosphotransporter proteins (AHPs). The phosphate group is then transferred to B-type response regulators (B-ARRs) like ARR11 and ARR10, activating downstream A-type ARRs to regulate the transcription of target genes [44]. Additionally, cytokinin signaling can be mediated through alternative pathways; for instance, in specific cells, signal perception may occur via the endoplasmic reticulum or the cell membrane, indicating cell-specific cytokinin signaling [45].

### 2.3. Role and Regulatory Mechanisms of Gibberellins

Gibberellins are a class of diterpenoid compounds characterized by a betulinene skeleton. Originally identified in the fungus Fusarium, they play crucial roles in promoting multiple aspects of plant growth and development, including seed germination, stem elongation, leaf expansion, flower initiation, and seed development [46,47,48,49]. Over one hundred types of gibberellins have been identified, with GA_1_, GA_3_, GA_4_, and GA_7_ being the primary ones. Among these, GA_3_ is the most commonly utilized gibberellin. Gibberellins play a crucial role in seed germination regulation through three main mechanisms. Firstly, they facilitate the degradation of the endosperm by weakening its structure, thereby releasing growth inhibitory substances located at the endosperm tip, which is a pivotal step in seed germination [50]. Secondly, gibberellins alleviate seed dormancy by stimulating the expression of specific genes (e.g., GA_20_ oxidase and GA_3_ oxidase) and elevating the levels of bioactive gibberellins (e.g., GA_4_ and GA_3_), thus promoting seed germination [51,52]. Furthermore, gibberellin counteracts the inhibitory effects of abscisic acid, facilitating seed germination. It also interacts with hormones like ethylene to jointly regulate the seed germination process [53]. Dong et al., 2022 found that in placing sweet cherry seeds in a medium containing GA_3_ could lead to a significant increase in seed germination [54]. In addition, gibberellin promotes cell wall loosening and cell elongation by inhibiting the activity of peroxidases in the cell wall, thereby reducing the formation of oxidatively coupled phenolic compounds [55]. Additionally, it stimulates cell division and increases cell numbers, working synergistically to promote stem elongation. In potato vitro culture, the application of GA_3_ significantly enhances the height of test-tube plantlets and accelerates seedling establishment [56]. Under non-tissue culture conditions, gibberellin can also regulate plant height and stem elongation in rice by influencing the expression of the PSRK2 gene [57]. Furthermore, gibberellin interacts with other hormones such as auxin to jointly regulate stem elongation. For instance, in cauliflower, gibberellin and auxin exhibit synergistic effects, although the role of gibberellin is generally more prominent [58].

The molecular mechanism underlying gibberellin’s regulation of plant growth primarily involves the signaling pathway. The core aspect of this signaling mechanism involves the alleviation of the inhibitory effect of the DELLA protein on plant growth through the interaction between the GID1 receptor and the DELLA protein. In the absence of gibberellin (GA), the DELLA protein suppresses plant growth and various developmental processes, such as seed dormancy and stem elongation. Conversely, in the presence of GA, it activates the downstream signaling pathway by binding to the GID1 receptor. This interaction triggers the degradation of the DELLA protein, thereby relieving its inhibitory effect and facilitating plant growth and development [59,60].

We must recognize that the application of exogenous PGRs in vitro culture does not function in a vacuum [61]. Their effects are ultimately executed through the plant’s innate endogenous hormonal systems. Exogenous PGRs can influence endogenous hormone levels by affecting their biosynthesis, metabolism, transport, and signaling. Therefore, the formation of callus and roots is ultimately the result of an orchestrated interplay between applied exogenous plant growth regulators and the preexisting endogenous hormonal milieu.

## 3. Application of Exogenous Plant Growth Regulators in Jujube In Vitro Culture

### 3.1. Research In Vitro Culture Rapid Propagation System of Jujube Plants

The first successful in vitro culture of jujube was established in 1978, when Shandong Agricultural College first used jujube endosperm for cultivation and obtained triploid plants [62]. Three years later, the Institute of Botany, Chinese Academy of Sciences, used in vitro culture methods to induce jujube embryos, producing embryo-like structures [63]. Using current-year young stem segments as explants, people developed an efficient organ culture protocol for ‘Tantan’ jujube, achieving successful plant regeneration [64]. Tao et al., 2015 used leaf, jujube bud, and dormant branch explants to establish the in vitro culture system for the “Mid-Autumn Crispy Jujube” variety [65]. Guo Y., 2020 established an adventitious shoot regeneration system using elite jujube cultivars, including ‘Junzao’, ‘Huizao’, and ‘Dongzao’ [66]. Three years later, research achieved in vitro induction of tetraploid and mixoploid adventitious shoots from leaf and axillary bud-containing stem explants in ‘Huizao’ jujube [67]. In the same year, others used stem segments from the current year’s jujube shoots of the triploid superior line T-185 as explant materials to establish an in vitro culture rapid propagation system for the triploid superior line [68]. This indicates that jujube in vitro culture has extended from basic embryo culture to multiple varieties and multiple explant systems, and is gradually focusing on polyploid breeding and innovative rapid propagation technologies. Additionally, corresponding in vitro culture rapid propagation systems have been established for ‘Pingguozao’, ‘Maoyezao’, ‘Dongzao’, ‘Huizao’, and ‘Junzao’.

There have been many studies in vitro culture of jujube. Regarding this, we have systematically summarized existing in vitro culture protocols for various jujube cultivars (Table 1) and in subsequent sections, a reference range of effective concentrations of plant growth regulators is proposed for each culture stage, which is applicable to most jujube varieties. The aim is to significantly reduce the time and trial-and-error costs during the preliminary experimental phase and to establish a solid foundation for subsequent system optimization and adjustment.

Overall, in vitro plant culture, auxins such as naphthaleneacetic acid (NAA) and indole-3-butyric acid (IBA) are widely employed, whereas indole-3-acetic acid (IAA) and 2,4-dichlorophenoxyacetic acid (2,4-D) are less frequently used. This preference is largely attributable to the superior chemical stability, pronounced physiological efficacy, favorable safety profile, and cost-effectiveness of NAA and IBA. In contrast, IAA, being naturally occurring, exhibits poor stability and is prone to degradation, limiting its practical application. Meanwhile, 2,4-D is generally restricted to specific purposes due to its potent herbicidal activity. Among cytokinins, 6-benzylaminopurine (6-BA) is preferred over zeatin (ZT) and kinetin (KT) owing to its greater stability, high efficacy in promoting cell division, and lower cost. Additionally, thidiazuron (TDZ), which exhibits exceptionally strong cytokinin-like activity, is often utilized as an auxiliary agent for recalcitrant plant species that are difficult to establish in vitro, typically at low concentrations. Within the gibberellin family, GA_3_ is the most extensively used member, based on a comprehensive evaluation of its biological effectiveness, cost, stability, and accessibility.

### 3.2. Application of Plant Growth Regulators in the Primary Induction Stage

The most commonly used medium for the primary cultivation of jujube is MS medium (Murashig and Shoog, 1962), with the molar ratio of N:P:K:Cl being approximately 48:1:16:2.4. However, there is evidence suggesting that the high nitrogen (N) and high chlorine (Cl) content in the MS medium promotes vegetative growth (cell expansion), but inhibits organogenesis, which can easily lead to glassy (hyperhydricity) and low-quality plants [61]. Nevertheless, despite its relatively high nitrogen-to-phosphorus ratio, MS medium remains the predominant choice for the in vitro culture of jujube. Since MS medium is widely used in the in vitro cultivation of jujube trees, while other formulations have been less frequently tested, it is still necessary to further verify whether the MS medium is the optimal choice.

The primary objective of initiation culture is to induce explants to form callus tissue or undergo direct differentiation into buds. Therefore, cytokinins and auxins are often used in combination at this stage, with the commonly used hormone combination being 6-BA 0.5~2.5 mg/L + NAA 0.1~1.0 mg/L. Different jujube varieties have certain differences in hormone requirements during primary culture. For example, Han J et al., 2014, added NAA 0.5 mg/L and 6-BA 2.0 mg/L to the MS medium for the anthers of Changhong jujube and found that the callus induction rate was over 94% [80]. Yang D.Z. et al., 2023 found that the suitable combination for the initiation of triploid superior line T-185 was 6-BA 2.0 mg/L + NAA 0.2 mg/L, which could increase the effective germination rate to 85% [68]. He establishment of the in vitro culture and rapid propagation system of ‘Zhongqiu Crispy Jujube’ demonstrated that a combination of 6-BA at 2.5 mg/L and NAA at 0.15 mg/L resulted in a high adventitious bud induction rate of 94% [65]. These findings confirm the efficacy of 6-BA and NAA in stimulating callus and bud development. The research indicates that the synergistic use of 6-BA and NAA generally enhances bud and adventitious bud formation; however, the optimal concentrations and ratios should be customized based on the jujube variety and explant type. Moreover, in some cases, a minor quantity of KT is introduced during the initial culture of certain varieties to prompt adventitious bud generation. In summary, for the initial culture of most jujube varieties, MS medium is recommended as the basal medium. The auxins NAA and IBA are suggested at concentrations ranging from 0.1 to 1.0 mg/L, while the cytokinin 6-BA is advised at a concentration of 0.5 to 2.5 mg/L.

### 3.3. Application of Exogenous Plant Growth Regulators in the Subculture Proliferation Stage

The purpose of subculture is to further proliferate and grow the buds obtained from the primary culture, which is specifically reflected in the level of proliferation coefficient. At this stage, it is necessary to timely adjust the optimal types and concentrations of plant growth regulators to promote the germination and growth of lateral buds, augment the clustered bud count, and judiciously incorporate auxins to sustain bud growth. In the subculture of jujube, the prevailing basic medium remains the MS medium, with commonly employed plant hormone combinations being 6-BA + IBA or 6-BA + IBA + TDZ. In vitro culture and rapid propagation of Ziziphus mauritiana, the suitable hormone combination for proliferation is 6-BA 1.2 mg/L + IBA 0.5 mg/L, and when proliferated to the 6th generation, the proliferation coefficient has reached 6.3 [71]. The study showed that the suitable hormone combination for subculture proliferation of triploid superior line Y3 is 6-BA 2.0 mg/L + IBA 0.2 mg/L + TDZ 0.1 mg/L, with a proliferation coefficient of 3.28 [85]. Chen R.H. et al., 2025 [76] used the stem segments with buds of Jun jujube as materials, and found that the suitable hormone combination for proliferation culture is 6-BA 1.0 mg/L + IBA 0.2 mg/L + TDZ 0.012 mg/L, with a proliferation coefficient of 4.26. Guo Y. 2020 [66] used the leaves of Jun jujube as materials, and the optimal plant growth regulator combination for adventitious bud proliferation is 6-BA 1.0 mg/L + IBA 0.2 mg/L + TDZ 0.005 mg/L, with a proliferation coefficient of 4.47. Qi X.Y. et al., 2010 [77] used the current-year jujube shoots of Jin jujube as materials and found that the suitable growth regulator combination for its proliferation is TDZ 0.01 mg/L + IBA 0.2 mg/L + 6-BA 1.0 mg/L, with a proliferation coefficient of 4.50. To sum up, in the subculture proliferation process of jujube, different jujube varieties have different suitable contents of plant growth regulators for proliferation. The proliferation effect of combining multiple plant growth regulators is better than that of using a single one, and 6-BA is often used in combination with IBA. In addition, TDZ can also be used in subculture proliferation. It has strong cytokinin activity and can improve the subculture proliferation coefficient, but excessively high concentration of TDZ may lead to significant differences in seedling vitrification. Therefore, only a small amount of TDZ needs to be added in subculture, generally less than 0.1 mg/L, and the concentration of TDZ applied varies greatly among different jujube varieties during subculture, so it needs to be adjusted according to specific conditions in application. In addition, a few studies also add GA3 in subculture, which can synergistically promote stem elongation with auxins. Zhou R.J. 2004 [73] added 0.5 mg/L GA_3_ to the subculture medium using winter jujube leaves as materials, which could significantly promote stem elongation, with the new shoots elongating by 4.20 cm, 1.54 cm longer than the control, and the proliferation coefficient was 2.02. The rational use of gibberellin can effectively regulate the morphogenesis, development process and stress resistance of tissue culture seedlings, and has important application value especially in the fields of rapid propagation, genetic transformation and artificial seed production.

In summary, for the subculture of most jujube varieties, The recommended concentration of auxins (such as NAA or IBA) ranges from 0.1 to 1.0 mg/L, while that of the cytokinin 6-BA is suggested to be between 0.5 and 2.5 mg/L. Additionally, the concentration of TDZ should be kept below 0.01 mg/L. MS medium is the most commonly used medium. However, the high ionic concentration in the MS medium limits its application in the proliferation cultivation of certain specific species. Therefore, further research is still needed to explore a dedicated proliferation medium suitable for jujube.

### 3.4. Application of Exogenous Plant Growth Regulators in the Rooting Culture Stage

Rooting culture is a crucial step in the rapid propagation of jujube through in vitro culture, where auxin substances play a dominant role. At this stage, the commonly used basic medium is 1/2 MS, and the plant growth regulators commonly used to promote rooting are naphthalene acetic acid (NAA) or indole butyric acid (IBA), which can also be used in combination. Studies have shown that the rooting rate of tissue-cultured seedlings of apple jujube reaches the highest at 91.6% under the condition of IBA 0.6 mg/L [69]. Xu J.R. et al., 2003 [71] compared the rooting effects of IBA, NAA used alone and in combination, and found that the rooting rate when using IBA alone was higher than that of the other two methods. Moreover, as the concentration increased, the rooting rate also increased. When IBA was 3.0 mg/L, the rooting rate reached the highest at 84.3%. When NAA was used alone, although the rooting rate was the highest at 82.3% when the concentration was 2.0 mg/L, the root system was not robust. In addition, a few studies have used IAA. Zhou R.J. 2004 [73] used Huanghua winter jujube as materials and found that the rooting rate was the highest at 87.1% when the concentration of IAA was 1.0 mg/L. In summary, rooting induction for most jujube varieties has predominantly been achieved using 1/2 MS medium, with growth regulator concentrations typically ranging from 0.5 to 3.0 mg/L. As can be seen from Table 1, the rooting effect of most jujube varieties with the combination of different auxins is better, but some varieties have better rooting effect with a single auxin. Thus, there are differences in the selection of hormones for root induction among different jujube varieties.

### 3.5. Analysis of Effects of Exogenous Plant Growth Regulators in Each Stage of Rapid Propagation of Jujube In Vitro Culture

**Figure 1:**
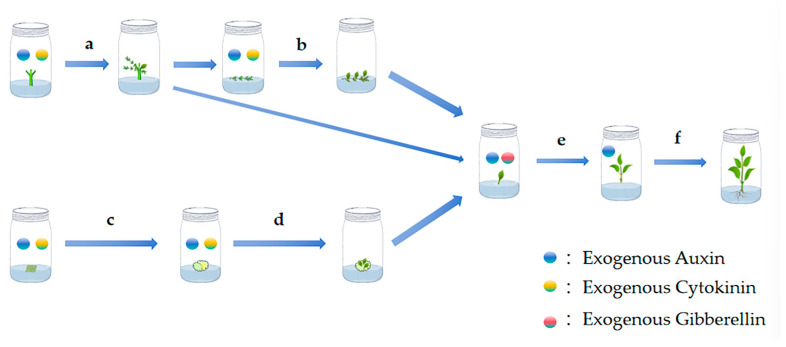
Application of plant growth regulators in the cultivation of Jujube organs, (**a**) First-generation induction, (**b**) Adventitious bud induction, (**c**) Callus induction, (**d**) Adventitious bud differentiation, (**e**) Subculture proliferation, (**f**) Rooting culture. Notes: The exogenous plant growth regulators added in the table exert their effects by stimulating the synthesis or signal transduction of endogenous auxins, thereby indirectly influencing the plants. It should be emphasized that the morphogenesis of plants is fundamentally regulated directly by their endogenous auxin levels and distribution.

Exogenous PGR can serve as effective inductor of endogenous PGR, which in turn plays a role in plant propagation, and can effectively promote the growth and development of jujube tissue by reasonably regulating their species and concentration, and realize the efficient and rapid propagation of jujube. At present, auxin is commonly used in all stages of jujube in vitro culture, cytokinin is commonly used in the stage of initiation culture, adventitious bud induction differentiation and subproliferation, and gibberellin is commonly used in the subproliferation stage (Figure 1). In the primary induction stage of jujube, the combination of auxin and cytokinin can induce callus formation (c) and differentiation budding (a, d). In primary induction, the explants will germinate and produce jujube hangers at the same time, which can be laid flat in a medium containing auxin and cytokinin to induce adventitious bud differentiation (b), and adventitious buds can be transferred to a medium containing auxin and gibberellin to promote bud proliferation and growth (e). When the vitro culture seedlings have 3–5 unfolded healthy leaves, strong stems and reach a height of about 3–5 cm, they can be transferred to rooting medium containing one or more auxins to promote rooting (f).

## 4. Prospects

From the perspective of domestic research on jujube fast breeding technology, although certain achievements have been made in this field, there are still many problems and challenges. For instance, challenges such as poor in vitro proliferation and inefficient vegetative growth regulation in jujube represent persistent obstacles in its tissue culture. Therefore, future research on the in vitro cultivation of jujube should prioritize the following areas: Firstly, research can delve into the molecular mechanism of the organogenesis pathway in jujube concerning the application of growth regulators. This includes investigating how exogenous plant growth regulators influence the endogenous hormone network to enhance plant growth. Secondly, there is a need to optimize the ratios of cytokinins (e.g., 6-BA), TDZ, and auxins (e.g., IBA, NAA). Developing sequential hormone combinations and determining the precise ratio of cytokinins and auxins are essential to stimulate the proliferation of jujube plants. The PGR ratio can also be refined for different varieties of jujube, different ploidy, and different stages of rapid propagation. For example, for jujube varieties that have already successfully established rapid propagation systems, refinement can be continued on the basis of the original to improve the survival rate; for those that have not yet established fast propagation systems in histoculture, a common medium formula can be selected for trial use, and then detailed research can be carried out according to the growth condition. When designing the hormones, it should be noted that the sum of the concentrations of each hormone should not exceed 3.0 mg/L, and the concentration of TDZ should not be more than 0.01 mg/L when it is used for the first time in the succeeding stage. Finally, from the perspective of the use of the culture medium, research has shown that a high nitrogen-to-phosphorus ratio can result in excessive vegetative growth, hyperhydricity, and poor shoot formation [61]. Nevertheless, despite its relatively high nitrogen-to-phosphorus ratio, MS medium remains the predominant choice for the in vitro culture of jujube. Therefore, we can system comparison of the effects of different basal media—particularly those with varied N and P ratios and anion compositions—on the in vitro regeneration of jujube, it will represent a highly valuable research direction to overcome current technical bottlenecks and optimize the regeneration system. In conclusion, the core of the future research on jujube tissue propagation should be to explore the new type of low-residue and high-efficiency plant growth regulators or their substitutes, to reduce the potential risks to the environment and the human body and to deeply probe the molecular, cellular and signaling levels. We should elucidate the precise mechanism of growth regulators in regulating the regeneration of jujube in vitro, especially somatic embryogenesis and organogenesis, and further optimize the fast propagation technology of jujube tissues to improve the propagation efficiency and quality of the seedlings, so as to push forward the development of jujube factory nursery to intelligent development. This will provide a solid theoretical foundation and precise control means for genetic improvement, efficient propagation and germplasm preservation of jujube.

## Figures and Tables

**Table 1:** Summary of the Application Research of Plant Growth Regulators in the Rapid Propagation of Jujube by in Vitro Culture.

Variety	Explant	Basic Culture Medium and Plant Growth Regulators	Result	Reference
Ping guo zao	Stem	P: 6-BA 6 mg/L + NAA 0.1 mg/L S: 6-BA 6 mg/L + NAA 0.1 mg/L R: IBA 0.6 mg/L	PC: 6.2 RR: 91.6% TSR: 98%	[69]
	Stem	P: 6-BA 1 mg/L + KT 0.5 mg/L S: 6-BA 1.5 mg/L + IBA 0.5 mg/L R: MS + 6-BA 1 mg/L + IAA 0.5 mg/L	Callus induction rate: 98% PC: 4–5 TSR: 96%	[70]
Mao ye zao	Stem	P: 6-BA 0.8 mg/L + IBA 0.4 mg/L S: 6-BA 1.2 mg/L + IBA 0.5 mg/L R: IBA 3 mg/L	Effective germination rate: 75.9% PC: 6.3 RR: 84.3%	[71]
Zhan hua Dong zao	Shoot tips	P: 6-BA 2 mg/L + KT 0.5 mg/L + NAA 0.1 mg/L S: 6-BA 2 mg/L + KT 0.5 mg/L + NAA 0.1 mg/L R: 6-BA 0.2 mg/L + IBA 0.8 mg/L	PC: 5.5 RR: 87.2% TSR: more than 80%	[72]
Huang hua Dong zao	Leaf	P: TDZ 1 mg/L + IBA 0.1~0.5 mg/L S: 6-BA 1 mg/L + KT 0.5 mg/L + IBA 0.1 mg/L R: IAA 1 mg/L	Leaf regeneration rate: 91.33% PC 3.64 RR: 87.10%	[73]
Lu bei Dong zao	Young branch tip	P: 6-BA 0.5 mg/L + KT 1 mg/L + IAA 0.5 mg/L S: 6-BA 2 mg/L + KT 0.5 mg/L + 2,4-D 0.2 mg/L R: IAA 0.5 mg/L + IBA 1 mg/L	Initial survival rate: 22.2% PC: 4 RR: 80% Average root number: 5 TSR: 90%	[74]
Dong zao	Leaf	P: TDZ 0.5 mg/L + NAA 0.1 mg/L S: TDZ 1 mg/L + NAA 0.3 mg/L	Adventitious bud differentiation coefficient: 2.77	[66]
Hui zao	Leaf	P: 1/2 MS + 6-BA 0.5 mg/L + 2,4-D 2 mg/L S: 6-BA 2.0 mg/L + IBA 0.5 mg/L + KT 0.5 mg/L R: NAA 0.5 mg/L	Callus induction rate: 96.67% PC: 3.06 RR: 95.56%	[75]
	Leaf	P: WPM + TDZ 1 mg/L + IBA 0.3 mg/L	Adventitious bud induction rate: 86.67% Average number of adventitious buds:1.99	[66]
	Stem	P: 6-BA 1 mg/L + IBA 0.2 mg/L S: 6-BA 1 mg/L + NAA 0.1 mg/L R: NAA 0.5 mg/L	Germination rate: 100% Proliferation rate: 83.33%	[67]
	Leaf	P: WPM + TDZ 1 mg/L + NAA 0.2 mg/L S: 6-BA 1 mg/L + IBA 0.2 mg/L + TDZ 0.005 mg/L R: IBA 0.5 mg/L + NAA 0.2 mg/L	Adventitious bud induction rate: 96.67% PC: 4.47 Average plant height: 2.00 cm RR: 100% Average number of roots: 11.23 Average root length: 0.49 cm	[66]
	Stem	P: 6-BA 1 mg/L + IBA 0.2 mg/L + TDZ 0.01 mg/L S: 6-BA 1 mg/L + IBA 0.2 mg/L + TDZ 0.012 mg/L R: IBA 0.6 mg/L	PC: 4.26 RR: 86.89% TSR: 96.30%	[76]
Jin zao	Shoot tips	P: 6-BA 0.5 mg/L S: TDZ 0.01 mg/L + IBA 0.2 mg/L + 6-BA 1 mg/L	PC: 4.50 Average plant height: 4.35 cm Number of new buds: 135	[77]
Zhong ning Xiao zao	Kernel of jujube	P: 6-BA 1 mg/L + NAA 1 mg/L S: KT 2 mg/L + IBA 1 mg/L + GA_3_ 1 mg/L R: IBA 0.8 mg/L + NAA 0.2 mg/L	Induction germination rate: 88.7% Bud differentiation rate: 66.7% Differentiation coefficient: 2–3 RR: 64%	[78]
	Shoot tips	P: 6-BA 2.5 mg/L + NAA 0.15 mg/L S: 6-BA 1.5 mg/L + NAA 0.5 mg/L R: NAA 0.15 mg/L	Induction rate of adventitious buds: 94.00% Average number of adventitious buds: 6.5 Average root count: 3.6	[65]
Lu zao 2 hao	Anther callus	P: 1/4 MS + NAA 0.3 mg/L + 2,4-D 1.5 mg/L S: WPM + KT 0.2 mg/L + IAA 0.5 mg/L + GA_3_ 1 mg/L	Differentiation rate of indeterminate buds: 100.0%.	[79]
Lu zao 6 hao	Anther callus	P: 1/4 MS + NAA 0.3 mg/L + 2,4-D 1.5 mg/L S: WPM + TDZ 0.1 mg/L + IAA 0.5 mg/L + GA_3_ 1 mg/L	Differentiation rate of indeterminate buds: 50.0%.	[79]
Chang hong zao	Anther callus	P: 6-BA 2 mg/L + NAA 0.05 mg/L S: 6-BA 1~2 mg/L + NAA 0.5 mg/L	Callus induction rate: over 94% Callus germination rate: above 60.0%.	[80]
La jiao zao	Leaf	P: 1/2 MS + TDZ 0.3 mg/L + IBA0.1 mg/L	Average number of adventitious buds regenerated per leaf: 5.4	[81]
Fu ping da zao	non-bud stem	P: 6-BA 1 mg/L + IBA 0.1 mg/L	Indeterminate bud regeneration rate: 85.42% Average number of buds sprouting: 3.23.	[82]
Jin si xiao zao	Anther callus	P: 1/2 MS + 2,4-D 1 mg/L S: 6-BA 1 mg/L + NAA 0.5 mg/L	Callus induction rate: 56.17% Callus budding rate: 20.36%	[80]
Wu he zao	Leaf	P: TDZ 1 mg/L + NAA 0.2 mg/L	Induction rate of adventitious buds: 52.2%	[83]
Le ling wu he jin si xiao zao	Shoot tips	P: modified MS + 6-BA 0.5 mg/L + IBA 0.2 mg/L S: modified MS + 6-BA 2 mg/L+ NAA 0.2 mg/L R: modified 1/2 MS + IAA 0.2 mg/L+ IBA 0.2 mg/L	Initiation survival rate of cultivation: 21.4% Differentiation coefficient: 4.2 RR: 91% Average number of roots: 4.1	[84]
T-185	Stem	P: 6-BA 2 mg/L + NAA 0.2 mg/L S: 6-BA 2 mg/L+ IBA0.2 mg/L + TDZ 0.1 mg/L R: IBA 2 mg/L + IAA 0.2 mg/L	Effective germination rate: 85% Average number of adventitious buds: 1.15 PC: 1.97 RR: 73.0%.	[68]

Note: (1) P stands for primary culture; S stands for subculture; R stands for rooting culture. (2) The culture medium employed was MS for P and S, and 1/2 MS for R, unless otherwise stated. (3) PC stands for Propagation Coefficient; RR stands for Rooting Rate; TSR stands for Transplant Survival Rate.
